# P-864. Solid organ transplantation as a potential driver for antibiotic resistance: development of an antibiogram relative to the time of transplantation in a tertiary care center in Japan

**DOI:** 10.1093/ofid/ofaf695.1072

**Published:** 2026-01-11

**Authors:** Satoshi Kitaura, Koh Okamoto, Miyuki Mizoguchi, Masahiko Ando, Minoru Ono, Eisuke Amiya, Masaru Hatano, Masaaki Sato, Nobuhisa Akamatsu, Kiyoshi Hasegawa, Daisuke Yamada, Haruki Kume, Yoshifumi Hamasaki, Takeya Tsutsumi

**Affiliations:** The University of Tokyo Hospital, Bunkyo-Ku, Tokyo, Japan; Institute of Science Tokyo, Bunkyo-ku, Tokyo, Japan; The University of Tokyo Hospital, Bunkyo-Ku, Tokyo, Japan; The University of Tokyo Hospital, Bunkyo-Ku, Tokyo, Japan; The University of Tokyo Hospital, Bunkyo-Ku, Tokyo, Japan; The University of Tokyo, Graduate School of Medicine, Bunkyo-Ku, Tokyo, Japan; The University of Tokyo Hospital, Bunkyo-Ku, Tokyo, Japan; Tokyo University Hospital, Bunkyo-ku, Tokyo, Japan; The University of Tokyo Hospital, Bunkyo-Ku, Tokyo, Japan; The University of Tokyo Hospital, Bunkyo-Ku, Tokyo, Japan; The University of Tokyo, Graduate School of Medicine, Bunkyo-Ku, Tokyo, Japan; The University of Tokyo, Graduate School of Medicine, Bunkyo-Ku, Tokyo, Japan; The University of Tokyo Hospital, Bunkyo-Ku, Tokyo, Japan; The University of Tokyo Hospital, Bunkyo-Ku, Tokyo, Japan

## Abstract

**Background:**

Solid organ transplant (SOT) recipients undergo dynamic changes in risk of infection pre- and post-SOT. While SOT recipients are at risk of acquiring drug-resistant organisms (DROs), whether time from SOT is associated with increased risk of DROs is uncertain. In this study, we evaluated the proportion of DROs according to different time periods relative to SOT.

**Methods:**

This is a single-center study performed at the University of Tokyo Hospital (UTH), Tokyo, Japan. Patients who underwent SOT at UTH from 1993/1 to 2022/12 were included. Susceptibility data collected between 2010 to 2022 were used to calculate percent susceptibility. The day of SOT was defined as Day 0 and time periods relative to SOT were defined. The percent susceptibility was calculated according to the Clinical and Laboratory Standards Institute guidelines. Target organisms were *Escherichia coli*, *Klebsiella pneumoniae*, *Pseudomonas aeruginosa*, *Staphylococcus aureus*, *Enterococcus faecalis*, and *E. faecium*. The percent susceptibility was compared between the pre- and post-SOT periods using Fisher’s exact test. A two-tailed p-value of < 0.05 was defined as significant.

**Results:**

A total of 1246 patients (182 heart, 135 lung, 862 liver, and 88 kidney) received SOT during this study period. Isolation of DROs among different time periods was infrequent ranging from 0.0 to 3.5% for carbapenem-resistant (CR) *E. coli*, CR *K. pneumoniae*, multidrug-resistant *P. aeruginosa*, and vancomycin-resistant *E. faecium*. Overall, the temporal susceptibility proportions relative to SOT were variable among different organisms and antibiotic combinations (Figure 1 and 2). Although susceptibility trends for *Enterococcus* species were less predictable, numerous antibiotics displayed decreased susceptibility within 360 days post-SOT while percent susceptibility beyond 360 days post-SOT did not differ significantly from that of pre-SOT.

**Conclusion:**

The risk for resistance may vary depending on microbe-antibiotic combinations and the relative time from SOT. An SOT-specific antibiogram adjusted to the peri-SOT timeline may address clinically relevant susceptibility patterns and help tailor empiric antimicrobial therapy.

**Disclosures:**

Koh Okamoto, MD, MS, PhD, Becton, Dickinson and Company: Honoraria|Shionogi: Honoraria|Terumo: Honoraria|Thermo Fisher Scientific: Honoraria

